# Morphological description and molecular characterization of *Heterospinus mccordi* n. gen. n. sp. (Acanthocephala: Polymorphidae) from cystacanths infecting a non-native crayfish host, *Procambarus clarkii* (Decapoda: Cambaridae), in South Carolina, USA

**DOI:** 10.1007/s11230-024-10195-8

**Published:** 2024-12-09

**Authors:** Gregory K. Rothman, Kristina M. Hill-Spanik, Graham A. Wagner, Michael R. Kendrick, Peter R. Kingsley-Smith, Isaure de Buron

**Affiliations:** 1 Marine Resources Research Institute, South Carolina Department of Natural Resources, Charleston, SC 29412 USA; 2 Department of Biology, College of Charleston, Charleston, SC 29412 USA

## Abstract

A new genus and species within the family Polymorphidae Meyer, 1931 were erected to accommodate cystacanths recovered from the mesentery of individuals from a non-native population of the red swamp crayfish *Procambarus clarkii* (Girard), collected from South Carolina (USA). Morphological characteristics of the specimens collected included in both sexes a spindle-shaped body with a slender hindtrunk, two fields of markedly different sized spines on the foretrunk, the presence of a middle row of smaller scythe-shaped hooks on a proboscis armed with 19–20 longitudinal rows of 14–16 hooks; and in males, six cement glands, absence of genital spines, and a digitiform and spinose bursa (observed inverted). Sequencing portions of both the mitochondrial cytochrome *c* oxidase I (COI) and large subunit ribosomal RNA genes was completed, followed by phylogenetic analysis of a concatenated alignment. Sequences from our specimens appeared in a clade with those of *Hexaglandula corynosoma* (Travassos, 1915) and *Ibirhynchus dimorpha* (Schmidt, 1973) but were 27% divergent from both using the COI marker. The genetic divergence of this parasite from other polymorphid genera, along with unique morphological features, justified erecting a new genus and new species. Herein we describe *Heterospinus mccordi* **n. gen.** **n. sp.** bringing the total number of genera within the family Polymorphidae to 16, and we emend and update the latest key that was provided for the genera within this family. This is the first record of polymorphids infecting *P. clarkii* outside of its native range. The definitive host remains unknown.

## Introduction

Acanthocephalans of the family Polymorphidae Meyer, 1931 primarily parasitize aquatic birds and marine mammals and use various crustaceans as intermediate hosts to complete their life cycle. A morphological diagnostic feature common to individuals in this family is the presence of some pattern of trunk spination (Yamaguti, 1963; Schmidt, 1973). Morphological characters used to differentiate among polymorphid species have primarily been trunk shape, the distribution of spines on the trunk and of hypodermal nuclei in the tegument, presence/absence of genital spines, the armature of the proboscis, and to a lesser extent, the number of cement glands (Schmidt, 1973; Presswell et al., 2020). Whereas such criteria can be convenient, morphological identification is typically difficult as many of these characters, supposedly diagnostic, have lost their boundaries over time, and many genera within the family Polymorphidae have overlapping morphological features and only very slight morphological distinctions (Schmidt, 1973; Aznar et al., 2006). This particularity of the family Polymorphidae has led to classification instability (García-Varela et al., 2013) with a history of recurrent reassessments of genera assignment of species within this family (e.g., Schmidt, 1973; 1975; Amin, 1992; Aznar et al., 2006; García-Varela & Pérez-Ponce de León, 2008; García-Varela et al., 2009). Hence, integrating information other than morphology is particularly relevant in the systematics of the family Polymorphidae. The usefulness of ecological parameters, such as the type of intermediate or definitive hosts, was demonstrated with the reintroduction of *Profilicollis* Meyer, 1931 and *Hexaglandula* Petrochenko, 1950, and erection of *Pseudocorynosoma* Aznar, Pérez-Ponce de León, Raga, 2006 (see Nickol et al., 1999; 2002; Aznar et al., 2006, respectively). More recently, the use of DNA sequencing has allowed clarification of the taxonomic arrangement and relationships among some genera of the family (García-Varela & Pérez-Ponce de León, 2008; García-Varela et al., 2009; 2011; 2013; Presswell et al., 2020; Ru et al., 2022), which is currently comprised of 15 accepted genera, including 11 for which molecular data are available (Presswell et al., 2020).

The red swamp crayfish *Procambarus clarkii* (Girard) is native to parts of the Gulf of Mexico coastal plain (Hobbs, 1989; Campos & Rodríguez-Almaraz, 1992) but is considered one of the World's most successful global invaders, primarily due to its trade as a commodity food source (Oficialdegui et al., 2019). *Procambarus clarkii* is known to have many detrimental impacts to environments where it is introduced (e.g., Twardochleb et al., 2013), including serving as an important host for pathogens, and in turn, contributing to declines in native biodiversity (Martín-Torrijos et al., 2021). In South Carolina (SC), USA, *P. clarkii* was introduced in the 1970s (Pomeroy & Kahl, 1987) and since its introduction has spread rapidly with concomitant declines in both the occurrence and diversity of native crayfishes (Kendrick et al., 2024).

The parasite community of *P. clarkii* has been well studied in its native range due to its importance in aquaculture (Edgerton et al., 2002; Longshaw, 2011), with the only reported acanthocephalan infection being by the polymorphid *Ibirhynchus dimorpha* (Schmidt, 1973) (formerly in *Southwellina* Witenberg, 1932) in Louisiana USA (Schmidt, 1973; García-Varela et al., 2011). Cystacanth infection in crayfish, also in Louisiana, was mentioned in Font (2007), but with no indication of the species of acanthocephalans encountered or of crayfish examined. Outside of its native range, acanthocephalan diversity associated with *P. clarkii* is not known, with one study in Hawaii examining specimens for infection by acanthocephalans but finding none (Font, 2007).

Examination of *P. clarkii* in coastal SC revealed infection by polymorphid cystacanths whose morphology and genetics did not match a described species in this family. Herein, we propose the erection of *Heterospinus mccordi* **n. gen.** **n. sp.** based on both morphological and molecular characteristics of cystacanths. This is the first record of a polymorphid infecting *P. clarkii* in a non-native locality.

## Materials and Methods

**Specimen collection**

Adult specimens of *P. clarkii* were collected in June 2023 (n = 4) and September 2023 (n = 57) via dip-net and baited minnow traps at Bear Swamp, a freshwater forested wetland located approximately 17 km WNW of Charleston, SC, USA (32.825448, -80.125018). Prior to dissections, individual crayfish were anesthetized for 30 min at -20 °C, and the cephalothorax and abdomen were incised dorsally to expose intestinal mesentery. After isolation in individual Petri dishes, cystacanths were removed from their cysts with fine needles and placed in tap water for 1 to 24 hours to allow for evagination of the proboscis and hindtrunk. Cystacanths were then fixed in 70% ethanol (EtOH) for 48 hours, and then preserved in 100% EtOH until further morphological and molecular analyses following the methods of Hernández-Orts et al. (2022).

**Morphological study**

*Light microscopy (LM)*

Specimens and hologenophores prepared for LM were first punctured with a fine needle and then stained and mounted using a variety of techniques. Eighteen specimens (two as proboscis only) were stained with acetocarmine, two specimens were stained with Gill’s hematoxylin and eosin-Y (H&E), and three specimens (two as proboscis only) were stained with eosin-Y only. All stained specimens were then slowly dehydrated in a gradient series of EtOH, cleared with methyl salicylate, and mounted in Canada or fir balsam. One specimen was directly mounted unstained in Hoyer’s medium and one other in lactophenol.

Observations were made with a DIC Olympus BX51 microscope equipped with a drawing tube. Line drawings were made by scanning and digitizing hand drawings, and measurements were made on digitized images using ImageJ (Schneider et al., 2012). Measurements were made in micrometers (unless otherwise stated) and are presented as the range followed by the mean in parentheses. Light micrographs were taken with a digital camera connected to the microscope. Type and voucher specimens were deposited at the Harold W. Manter Laboratory (HWML), University of Nebraska, Lincoln, Nebraska, USA and the Smithsonian National Museum of Natural History (USNM), Washington DC, USA.

*Scanning electron microscopy (SEM)*

Two cystacanths were processed for SEM. Three holes were punctured with a fine needle in each specimen that were then fully dehydrated in two baths of 100% EtOH, and chemically dried in hexamethyldisilazane (Sigma-Aldrich, St. Louis, MO, USA) overnight. Specimens were then mounted on conductive double-sided carbon tape, sputter-coated in gold at 2 atm and 10 mA for 40 s and observed using a Hitachi TM-1000 SEM at 15kV.

*Histology*

Seven specimens were prepared for histological processing. To facilitate and optimize embedding, the proboscis, neck, and upper part of the trunk were severed from the rest of the trunk at approximately the mid-level of the proboscis receptacle on four of the specimens. The trunk was subsequently stained with eosin-Y prior to being processed for histology following standard procedures. Serial sections (7 µm thick) were stained with H&E and coverslipped in Cytoseal XYL (Richard-Allan Scientific, Kalamazoo, MI, USA). Proboscides of specimens were stained independently and used in the morphological analysis (see above).

*Voucher specimen examination*

Voucher specimens requested from the HWML and the USNM were examined. These included juveniles and adults of *Southwellina hispida* (Van Cleave, 1925) from the brown pelican, *Pelecanus occidentalis* L. (HWML 34897, 34898, 34902, 34903; Schmidt’s collection) and the roseate spoonbill, *Platalea ajaja* L. (USNM 1378324; Sepúlveda et al., 1994), and cystacanths of *S. hispida* from the gobiid longjaw mudsucker, *Gillichthys mirabilis* Cooper (photovoucher HWML 34528; Amin et al., 2022); one adult and one cystacanth of *S. macracanthus* (Ward & Winter, 1952) from the yellow-billed tern, *Sternula superciliaris* (Vieillot) (HWML 34527) and from the sand seatrout, *Cynoscion arenarius* Ginsburg (HWML 34528), respectively (Schmidt’s collection). Furthermore, we examined adults and juveniles of unidentified polymorphids (labeled *Arhythmorhynchus* (*Southwellina*)) from the whooping crane, *Grus americana* (L.) (USNM 1380940; Spalding et al., 1996) and from the little blue heron, *Egretta caerulea* (L.) (USNM 1379584; Sepúlveda et al., 1994 and USNM 1386160; Dronen & Chen, 2002) in case they could be adults of the species we describe herein.

**Molecular study**

*DNA Extraction*

A small triangular piece of tegument from newly collected cystacanths (n = 2) was removed from the dorsal side of each specimen. Additionally, 4 paragenophores were processed. Genomic DNA was extracted from tissue using a DNeasy Blood and Tissue kit (Qiagen, Valencia, CA, USA) according to the manufacturer’s instructions. DNA was then concentrated to ~100 µl using an Eppendorf VacufugePlus (Hamburg, Germany) prior to amplification.

*Polymerase chain reaction (PCR)*

PCR targeted partial fragments of the mitochondrial cytochrome *c* oxidase subunit I (COI) and nuclear large subunit ribosomal RNA (LSU rRNA) genes of the acanthocephalan. COI fragments were amplified using the primers jgLCO1490 (5′-GGTCAACAAATCATAAAGATATTGG-3′) and jgHCO2198 (5′-TAAACTTCAGGGTGACCAAAAAATCA-3′, Geller et al., 2013). A 25-μl total reaction contained 1x Promega GoTaq Flexi PCR Buffer (Madison, WI, USA), 2 mM MgCl_2_, 0.25x Rediload^TM^ (Invitrogen, Waltham, MA, USA), 0.2 mM dNTPs, each primer at 0.3 μM, 1 U Promega GoTaq® DNA polymerase, and 3 μl template DNA. Cycling conditions were as follows: denaturation at 95 ℃ for 5 min, 35 cycles of 95 ℃ for 30 s, annealing at 48 ℃ for 30 s, extension at 72 ℃ for 45 s, and final extension at 72 ℃ for 5 min. Partial fragments of the LSU rRNA gene were amplified using primers from García-Varela & Nadler (2006) including LSU amplicon 1 (forward 5′-CAAGTACCGTGAGGGAAAGTTGC-3′, reverse 5′-CAGCTATCCTGAGGGAAAC-3′) and amplicon 2 (forward 5′- ACCCGAAAGATGGTGAACTATG-3′, reverse 5′-CTTCTCCAAC(T/G)TCAGTCTTCAA-3′). A 25-μl total reaction contained the same reagents and concentrations as above except for 1 µM of each primer was used. PCR cycling conditions were as follows: denaturation at 94 ℃ for 3 min, 35 cycles of 94 ℃ for 1 min, annealing at 56 ℃ (amplicon 1) and 54 ℃ (amplicon 2) for 1 min, extension at 72 ℃ for 1 min, and a final extension at 72 ℃ for 7 min. All products were electrophoresed on 1% agarose gels (100 V, 30 min) that were pre-stained with GelRed (Biotium, Inc., Hayward, CA, USA) and visualized under UV light. PCR products were cleaned using ExoSAP-IT^TM^ (Affymetrix, Santa Clara, CA, USA) following the manufacturer’s instructions except that the reagent was diluted (1:10) and incubated at 80 ℃ for 30 min instead of 15 min. Products (n = 6 for COI, n = 1 for LSU) were sent to Eurofins MWG Operon LLC (Louisville, KY, USA) for direct bi-directional sequencing using the same primers as above.

*Alignments and phylogenetic analyses*

Contiguous sequences were assembled, and base-calling differences were resolved using Sequencher v 5.4 (Gene Codes Corporation, Ann Arbor, MI, USA). COI sequences from our specimens (n = 6) were aligned with one another using ClustalW (Thompson et al., 1994) using default parameters. The longest COI sequence was then aligned with those from polymorphid specimens in GenBank using ClustalW (Thompson et al., 1994) in MEGA11 (Tamura et al., 2021). The LSU sequence from our specimen (n = 1) was aligned using MUSCLE (Edgar, 2004), also in MEGA11 (Tamura et al., 2021). Sequences of a *Centrorhynchus* sp. were chosen as the outgroup based on previous studies, including García-Varela et al. (2013) and Presswell et al. (2018). For the LSU alignment, NGPhylogeny.fr webservice (Lemoine et al., 2019) was also used to implement Gblocks 0.91.1 (Castresana, 2000) for selection of conserved regions. All alignments were trimmed to eliminate terminal gaps prior to analysis. COI (586 base pairs (bp)) and LSU alignments (827 bp) were then concatenated, producing a 1,413-bp alignment used for phylogenetic and distance analyses. Maximum parsimony (MP) analysis (1,000 bootstrap replicates with 100 random additions) based on 1,362 informative characters was conducted in MEGA11 (Tamura et al., 2021) using the subtree-pruning-regrafting (SPR) algorithm (Nei & Kumar, 2000). Bayesian analysis was performed using MrBayes 3.2.7_0 (Huelsenbeck & Ronquist, 2001) accessed via NGPhylogeny.fr (Lemoine et al., 2019) with the GTR + I + G model, which was selected using jModeltest 2.1.9 (Darriba et al., 2012). Ten thousand trees were produced (2 runs, 4 Markov chains, 10 million MCMC generations, sample frequency = 1,000), with 25% removed as burn-in. The 50% majority-rule consensus tree resulting from the Bayesian analysis was visualized and edited in iTOL 6.9.1 (Interactive Tree of Life; Letunic & Bork, 2024); nodes with posterior probabilities (PP) < 0.90 were condensed further. Inkscape 0.92 (www.inkscape.org) was then used to add MP bootstrap support values to the nodes. COI p-distance calculations were performed in MEGA11 (Tamura et al., 2021). Sequences from this study were deposited into GenBank as accession numbers PQ219552-PQ219557 (COI) and PQ285816 (LSU).

## Results

Order Polymorphida Petrochenko, 1956.

Family Polymorphidae Meyer, 1931.

*Heterospinus* **n. gen.**

Diagnosis

Trunk spindle-shaped with marked constriction at point of evagination of hindtrunk (possibly due to the immaturity of the specimens). Foretrunk with two anterior spinose fields, dorsal hump at mid region. Hindtrunk tubular, slender, about half the size of foretrunk, fully invaginated in most cystacanths. Two fields of complete circular rows of spines occupying less than 25% of foretrunk with distinctly larger spines in anterior field than in posterior field; anterior field directly posterior to neck separated from posterior field by narrow but distinct bare zone; anterior field well-organized with greater number of spinose rows ventrally, occasionally disorganized posteriorly; posterior field characterized by unorganized spines anteriorly, more organized towards posterior rows; size of spines generally decreasing posteriorly in both fields. Tegument of foretrunk thick, with numerous hypodermal nuclei, fragmented; thicker between posterior spinose field and constriction of hindtrunk. Tegument of hindtrunk thin, non-nucleated. Neck short, ca. half-length of proboscis. Proboscis cylindrical, armed with numerous longitudinal rows of hooks rooted anteriorly and rootless posteriorly; first rootless hook smallest, scythe-shaped; slight swelling ca. mid-level at point of transition from rooted to rootless hooks. Apical ganglion absent. Proboscis receptacle inserted at base of proboscis; double-walled; distal end single-walled. Cerebral ganglion located ca. mid-level of proboscis receptacle. Lemnisci leaf-shaped, barely discernible, originating at base of proboscis and ending near base of second spinose region. Testes round to ovoid, in tandem; position in body cavity varied but often near mid-level of foretrunk in cystacanths. Cement glands 6, tubular, distal ends over posterior testis. Saefftigen’s pouch prominent, located in foretrunk. Bursa (only observed inverted) muscular, spinose, and apparently digitiform. Gonopore terminal in male. Genital spines absent. Genitalia, gonopore and genital spines not observed in female cystacanths.

Type species: *Heterospinus mccordi* **n. sp.**

ZooBank registration: The Life Science Identifier (LSID) for *Heterospinus*** n. gen.** is urn:lsid:zoobank.org:act:E0C92E6C-B97F-4079-9154-4A02F9D5747D

Etymology: The name of the genus is in reference to the markedly different sizes of spines between the two spinose fields on the foretrunk of both male and female individuals.

**Fig. 1:**
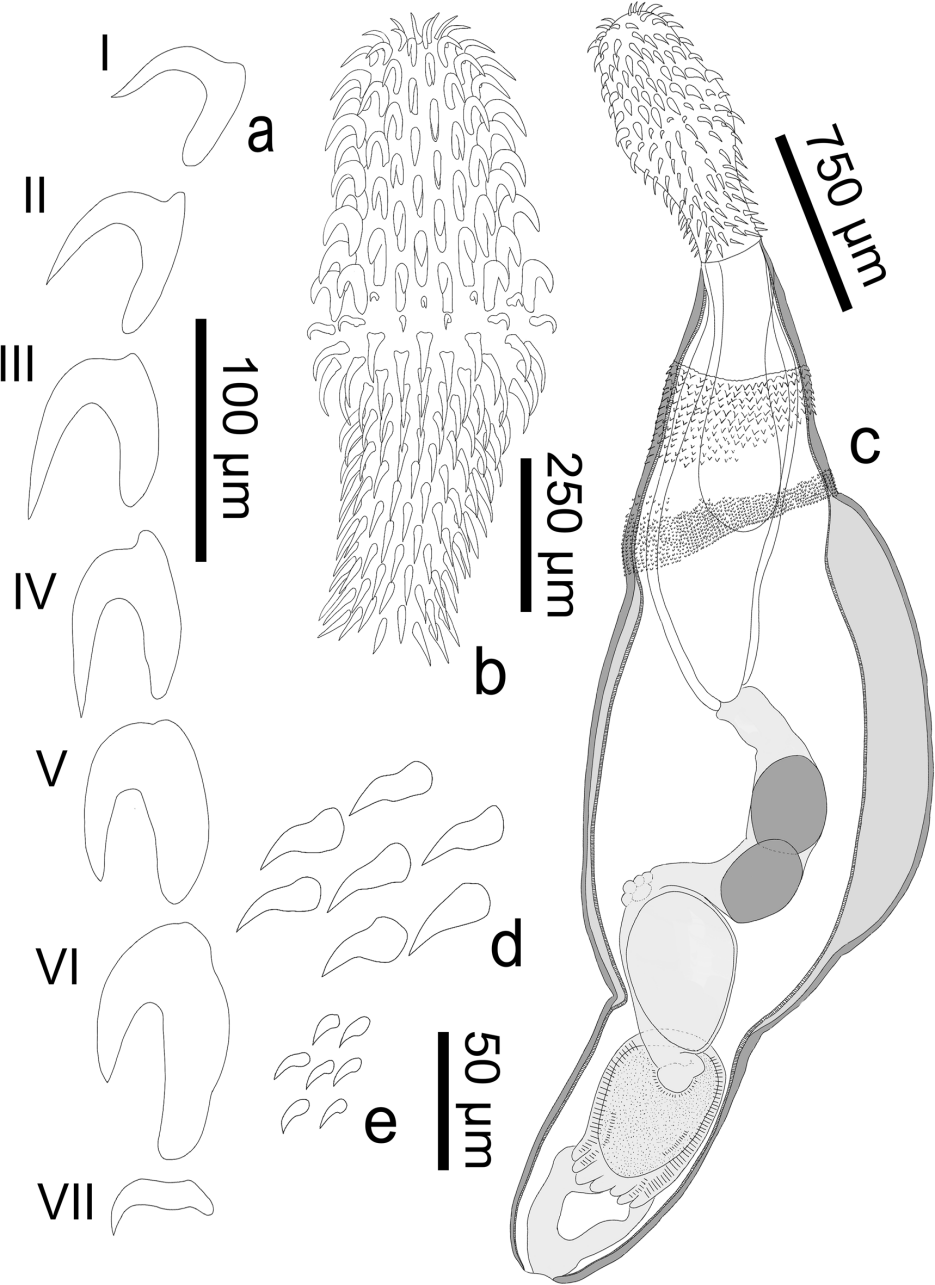
Line drawings of cystacanth male holotype of *Heterospinus mccordi* **n. gen.** **n. sp.** ex *Procambarus clarkii.* a, rooted proboscis hooks I-VI and the characteristic smaller scythe-shaped rootless hook VII; b, proboscis; c, whole individual; d, subset of spines from foretrunk anterior spinose field; e, subset of spines from foretrunk posterior spinose field

**Fig. 2:**
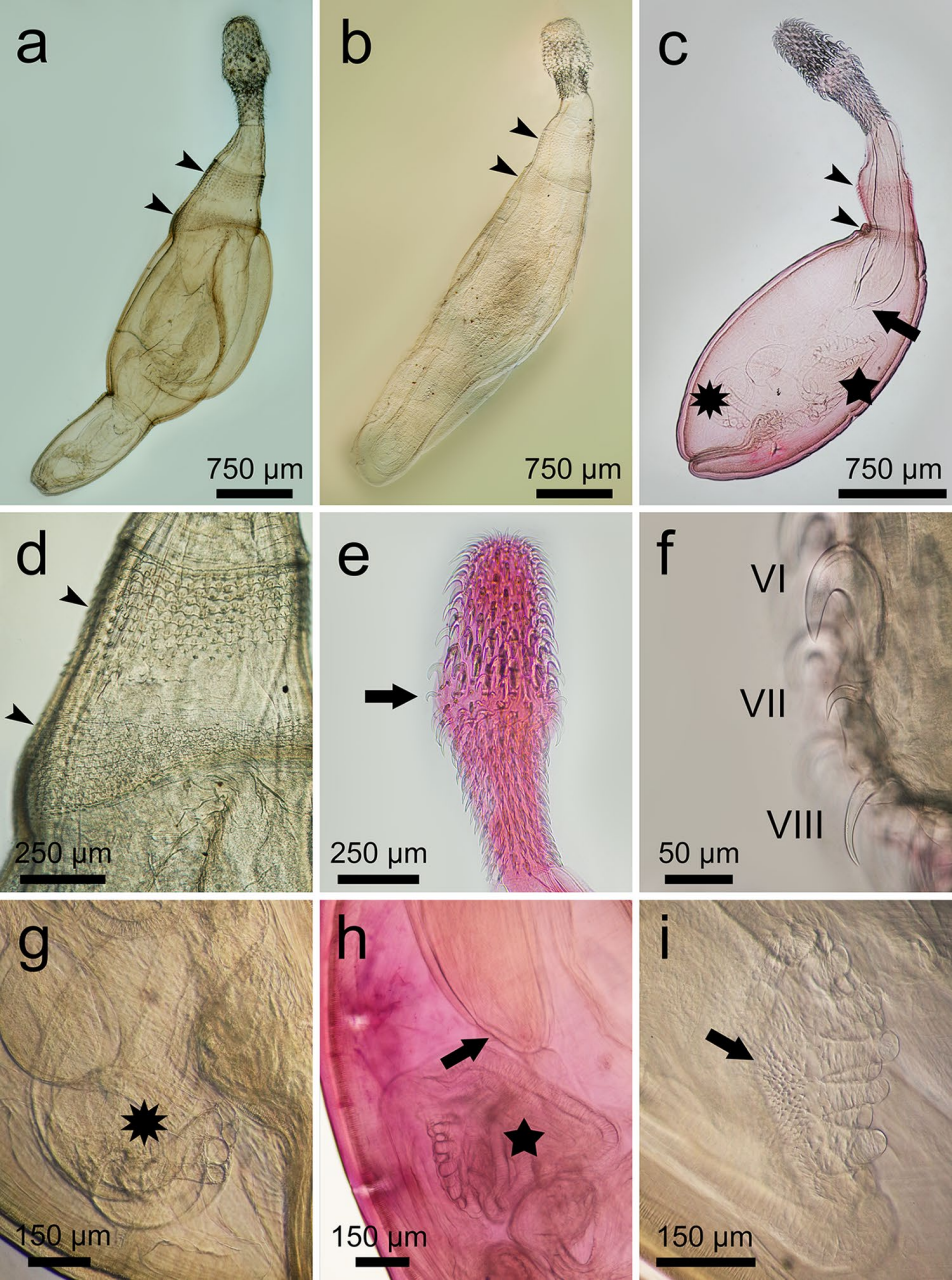
Whole mounts of cystacanths of *Heterospinus mccordi* **n. gen.** **n. sp.** ex *Procambarus clarkii*. a, composite of the male holotype showing exerted hindtrunk and the two spinose fields (arrowheads); b, composite of female with exerted hindtrunk showing position of the two spinose fields (arrowheads); c, male paratype with inverted hindtrunk. Note the single-walled distal end of the proboscis receptacle (arrow), the thickening of tegument below second field of spines (arrowheads), the apparently digitiform bursa (star), and the cement glands (10-point star). Note thick tegument of foretrunk starting below the posteriormost field of spines; d, detail of the two spinose fields on the foretrunk (arrowheads); e, proboscis of male showing typical swelling and smaller scythe-shaped hook ca. mid-level (arrow); f, proboscis hooks VI (with root), VII (scythe-shaped), VIII (rootless); g, foretrunk showing cement glands (10-point star); h, digitiform bursa (star) and single-walled distal end of proboscis receptacle (arrow); i, higher magnification of bursa showing digits and spines (arrow)

**Fig. 3:**
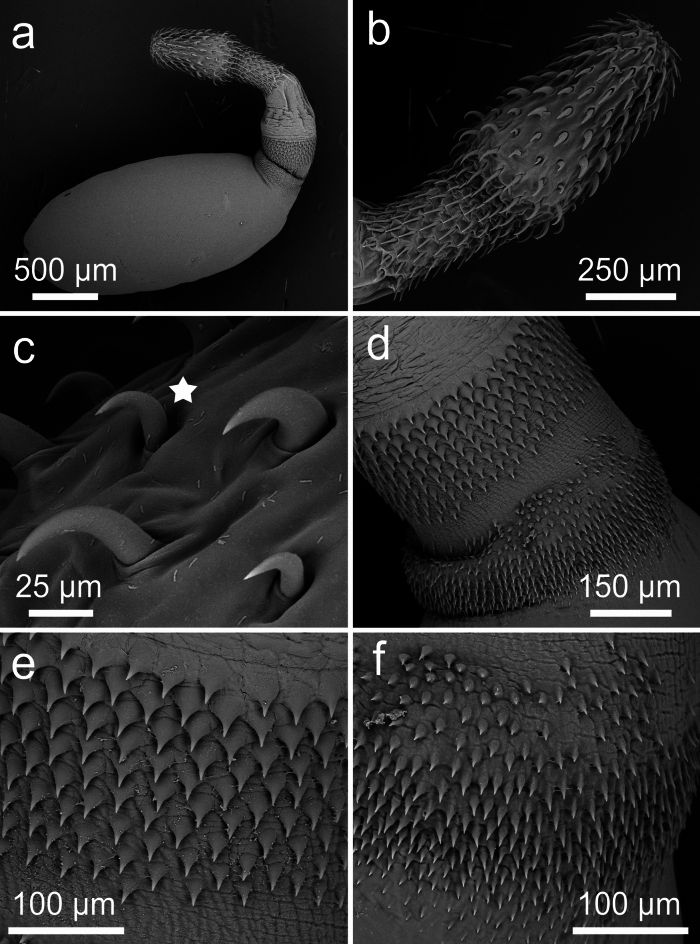
SEM of cystacanths of *Heterospinus mccordi* **n. gen n. sp.** ex *Procambarus clarkii.* a, cystacanth with invaginated hindtrunk; b, proboscis; c, scythe-shaped hook VII on proboscis (star); d, anterior and posterior foretrunk fields of spines; e, anteriormost field of larger spines; f, posteriormost field of smaller spines

**Fig. 4:**
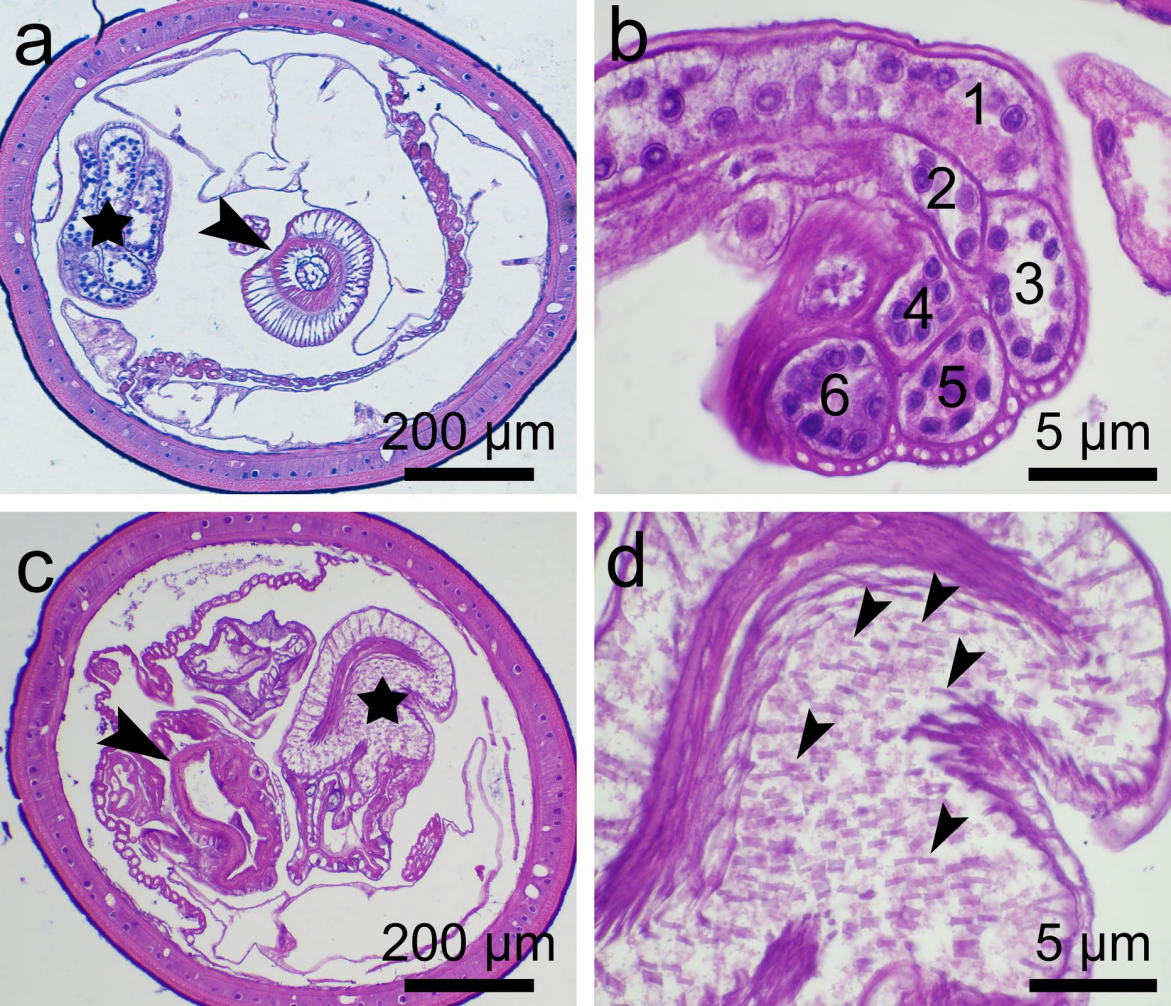
Histological sections of male cystacanths of *Heterospinus mccordi* **n. gen. n. sp.** ex *Procambarus clarkii*. a, anterior portion of midtrunk showing distal single-walled proboscis receptacle (arrowhead), nucleated thick tegument, and cement glands (star); b, cement glands (numbered) with fragmented nuclei; c, posterior section of mid foretrunk with invaginated hindtrunk (arrowhead) showing an anucleated and thin tegument and muscular copulatory bursa (star); d, copulatory bursa showing spines (arrowheads). H&E

*Heterospinus mccordi* **n. gen.** **n. sp.** (Figs. 1, 2, 3, 4)

Type host (intermediate host): *Procambarus clarkii* (Girard)

Definitive host: Unknown

Type locality: Bear Swamp, South Carolina, USA (32.825448, -80.125018)

Type material: Holotype HWML-217875 (male cystacanth); 4 paratypes (3 male and 1 female cystacanths) HWML-217876, 217877; USNM 1739945, 1739946 (2 males); 4 hologenophores (2 male and 2 female cystacanths) HWML-217878, 217879; USNM 1739949, 1739950; 5 vouchers (including 2 isolated proboscides) HWML-217880-217882; USNM 1739947, 1739948.

Site of infection: Hemocoel, attached to intestinal mesentery

Infection parameters: Prevalence: 18% (11 of 61 individuals of *P. clarkii* examined); mean intensity: 4.2 (47 cystacanths recovered from 11 individuals); range: 1-11

Representative DNA sequences: GenBank PQ285816 (LSU), PQ219552-PQ219557 (COI)

ZooBank registration: The Life Science Identifier (LSID) for *Heterospinus mccordi* **n. gen.** **n. sp.** is urn:lsid:zoobank.org:act:3E621722-234B-4A3C-A269-27B43F8E60C2

Etymology: The specific name recognizes long-serving South Carolina Department of Natural Resources Wildlife Biologist and naturalist John William “Billy” McCord who recorded, on multiple accounts, the presence of the non-native *P. clarkii* within coastal SC. Without his contributions to our understanding of the distribution of *P. clarkii* in Bear Swamp and his long-standing concerns related to the ecological impacts of this species on native crayfishes of the southeastern United States, this novel acanthocephalan genus and species may have gone undetected.

### Description (Figs. 1–4; Table 1)

General (based on 26 cystacanths: 20 whole specimens stained and mounted in balsam, 2 prepared for SEM, 4 isolated proboscides stained and mounted in balsam with respective trunks sectioned for histology). Tegument pinkish-orange in color *in vivo*. Sexual dimorphism slight, limited to size of hooks on proboscis and receptacle width. Trunk spindle-shaped with marked constriction separating foretrunk and hindtrunk (albeit a possible reflection of specimens’ immaturity). Foretrunk 1.96-2.56 (2.21) mm long, slightly enlarged at mid-level, 0.94-1.37 (1.16) mm at largest width; tapering anteriorly towards neck; thick tegument with numerous fragmented hypodermal nuclei; armed with two fields of circular rows of spines of distinctively different sizes, separated by thin bare zone. Spines in anteriormost field 26-33 (29) long (SEM), well organized anteriorly, with 7-9 rows dorsally extending to 10-12 rows ventrally. Spines in posteriormost field 13-19 (16) long (SEM), disorganized peripherally, with 12-15 circular rows. Neck short, 248-449 (299) long by 200-290 (250) wide at distal end and 283-484 (360) at proximal end. Proboscis receptacle 1019-1747 (1388) long, 321-552 (402) at widest point; single walled at distal end. Proboscis 842-1142 (938) long, 230-390 (318) at widest point, with 19-20 longitudinal rows of 6 anterior rooted hooks and 8-10 posterior rootless hooks; swollen ca. mid-level at transition from rooted to rootless hooks. Hooks I-VI progressively increasing in size; roots increasing in size approaching swollen region; hooks I-III with blade and root lengths generally equal; hooks IV-VI with roots generally longer and stronger than blades. Hook VII smaller, scythe-shaped; rootless hooks blade lengths slightly decreasing posteriorly.

**Table 1:** *Heterospinus mccordi* **n. gen. n. sp.** ex *Procambarus clarkii.* Proboscis hook blade and root lengths in µm, listed as range (mean); measurements made on four mounted male and three female cystacanths. n = number of hooks measured

Hook	Male				Female			
	Blade Length	n	Root Length	n	Blade Length	n	Rooth Length	n
I	23–44 (32)	7	21–48 (37)	5	43–58 (48)	3	40–48 (44)	2
II	46–72 (57)	9	30–67 (54)	6	41–86 (65)	10	49–66 (59)	6
III	60–76 (70)	10	59–69 (65)	6	66–92 (82)	6	59–75 (67)	2
IV	59–84 (66)	10	62–68 (64)	6	67–89 (82)	5	65–84 (71)	3
V	60–71 (65)	11	62–96 (85)	7	73–91 (78)	5	71–95 (82)	3
VI	39–64 (54)	10	72–106 (88)	9	61–91 (78)	7	87–125 (106)	5
VII	19–33 (26)	12	–	–	28–43 (34)	6	–	–
VIII	35–89 (68)	5	–	–	74–84 (79)	2	–	–
IX	62–96 (81)	5	–	–	70–95 (83)	3	–	–
X	55–84 (73)	5	–	–	54–85 (70)	2	–	–
XI	45–71 (59)	5	–	–	62–72 (67)	2	–	–
XII	47–73 (58)	5	–	–	63–77 (70)	2	–	–
XIII	52–69 (60)	5	–	–	63–63 (63)	2	–	–
XIV	53–69 (60)	5	–	–	60–68 (64)	2	–	–
XV	46–72 (61)	5	–	–	56–57 (56)	2	–	–
XVI	49–66 (58)	4	–	–	–	–	–	–

*Male* (based on 17 immature specimens: one cystacanth with hindtrunk fully exerted mounted in Hoyer’s medium, 14 cystacanths with hindtrunk invaginated mounted in balsam, 2 cystacanths with only the proboscis mounted): With general characteristics. Hooks slightly smaller than in females. Proboscis receptacle slightly narrower than in females, 321-450 (386) at widest point. Testes round to ovoid, equal in size, 300-400 (330) long by 240–440 (310) at largest diameter. Saefftigen’s pouch 600 long by 400 wide near constriction of hindtrunk. Tubular cement glands 6, with distal ends over posterior testis. Hindtrunk tubular 1130 long by 630 wide, with thin anucleated tegument; copulatory bursa muscular, spinose, apparently digitiform (viewed inverted). Gonopore terminal. Genital spines absent.

*Female* (based on 7 cystacanths: 5 with proboscis evaginated but hindtrunk not exerted, one with hindtrunk fully exerted, one with only proboscis mounted). With general characteristics same as males. Hooks on proboscis slightly larger than in males except for hooks V and VI that are substantially larger. Proboscis receptacle slightly wider than in males, 353-552 (447) at widest point. Genitalia and gonopore not observed. Eggs unknown.

Remarks

The erection of the new genus and new species to accommodate our specimens is based on morphological and molecular analyses. Following the key of Presswell et al. (2020), specimens collected from *P. clarkii* in SC most closely resemble *Ibirhynchus* García-Varela, Pérez-Ponce de León, Aznar & Nadler, 2011, *Southwellina* Witenberg, 1932, *Polymorphus* Lühe, 1911, and *Hexaglandula* Petrochenko, 1950 in having a trunk that is spinose, spindle-shaped, not elongated and without obvious bulbous swelling, a short neck, and no genital spines. Our specimens, however, differ from each of these four genera in either having a cylindrical proboscis with a mid-level swelling in both sexes (*Polymorphus* and female *Ibirhynchus* have an ovoid proboscis) and/or two fields of spines in both sexes (*Hexaglandula* and *Polymorphus* have one field and only males of *Ibirhynchus* have two fields) and/or having six cement glands (*Ibirhynchus*, *Southwellina*, and *Polymorphus* have four). Having only one field of spines, an ovoid proboscis (in one or both sexes), and four cement glands sets *Polymorphus* and *Ibirhynchus* apart from the new genus. Whereas our specimens resemble *Hexaglandula* in having a similar cylindrical proboscis with a mid-level swelling in both sexes and six cement glands, they differ from this genus in having two spinose fields on their foretrunk and an anucleate hindtrunk (*Hexaglandula* has one field of spines and hypodermal nuclei throughout the entire trunk). In having a similar proboscis shape, fragmented hypodermal nuclei restricted to the foretrunk, and two fields of spines on their foretrunk, our specimens resemble most closely *Southwellina*; however, our specimens differ from *Southwellina* in the number of cement glands (six vs four in *Southwellina*) and in their trunk spines having very different sizes in both fields (spines are the same size in *Southwellina*). Examination of voucher specimens of *S. macracanthus* and *S. hispida* confirmed that spines are the same size in both fields on the foretrunk of males and females and significantly larger than in our specimens.

Therefore, while the newly collected specimens have morphological features that overlap with these four genera, which then cannot be diagnostic when singled out, the one feature that strikingly separates the new genus from these genera is the marked difference in spine size between the two spinose fields on the foretrunk of both males and females. In this regard, we re-visited the description of *Hemiechinosoma* Petrochenko & Smogorjevskaia, 1962 as Schmidt (1973) did not mention spine size when he synonymized this genus with *Southwellina*. From the figures of Petrochenko & Smogorjevskaia (1962), we could verify that spines were of similar size in both fields. Importantly, specimens collected herein have additional characters that set them apart, including one small scythe-shaped hook at the transition between rooted and rootless hooks on the proboscis, a double-walled proboscis receptacle that becomes single-walled prior to its distal extremity, and a spinose and apparently digitiform bursa in males. These features, which may be of diagnostic value are, however, less conspicuous and difficult to verify on most voucher specimens (although *Southwellina hispida* HMWL 34902 clearly has a double-walled proboscis receptacle to the very distal end).

**Fig. 5:**
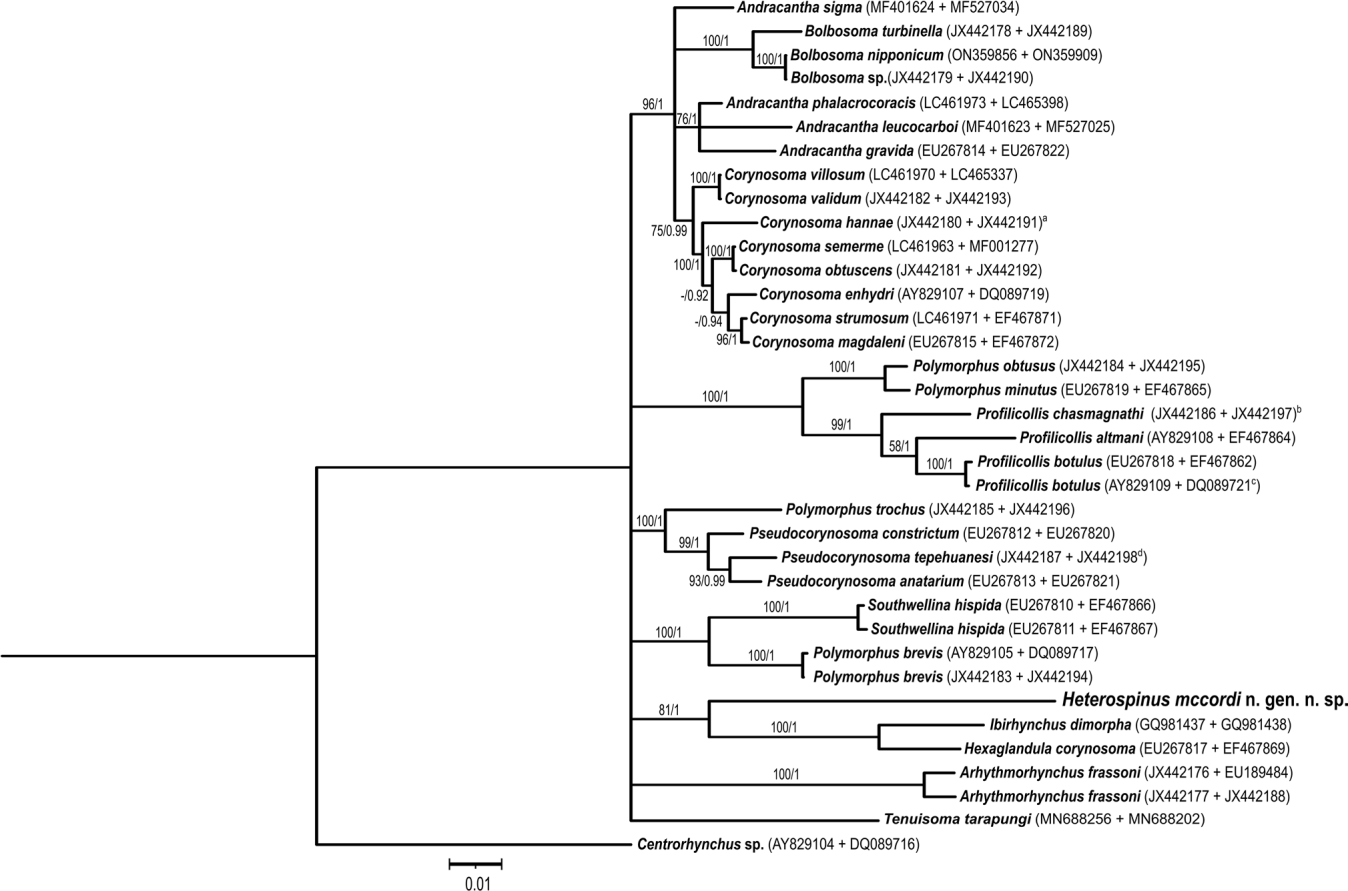
Bayesian 50% majority rule consensus tree based on a concatenated alignment of partial large subunit ribosomal RNA (LSU rRNA) and mitochondrial cytochrome *c* oxidase I (COI) gene sequences of polymorphids found in GenBank along with sequences from *Heterospinus mccordi* **n. gen.** **n. sp.** generated in this study. Numbers at nodes indicate bootstrap support values resulting from the maximum parsimony followed by posterior probabilities from Bayesian inference analyses. GenBank accession numbers of LSU and COI sequences, respectively, are in parentheses following the names of each taxon.

Significantly, phylogenetic analysis of concatenated LSU and COI data showed that sequences from our specimens were in a monophyletic clade with sequences from *I. dimorpha* (Schmidt, 1973) and *H. corynosoma* (Travassos, 1915) (MP bootstrap support = 81, Bayesian PP = 1; Fig. 5). COI sequence from our specimens and those from these two species were 27% different based on a 586-bp alignment (Table 2), which is within the range of intergeneric distances reported between other polymorphid genera (22-30% based on 655-bp alignment; García-Varela & Pérez-Ponce de León, 2008) and justifies the erection of a new genus. Intraspecific variation among the COI sequences was only 0.7-1.6% (n = 6, 513-bp alignment). Lastly, *I. dimorpha* and *H. corynosoma* also use decapods for intermediate hosts (Schmidt, 1973; Nickol et al., 2002; Guillén-Hernández et al., 2008), which further supports the phylogenetic relationship of the species in this clade. It may be of further interest to note that species in this clade may be specialists for their definitive hosts; this was demonstrated for *H. corynosoma* that appears to infect only the yellow crowned night heron, *Nyctanassa violacea* (L.) (García-Varela et al., 2023); *I. dimorpha* has only been reported from the white ibis, *Eudocimus albus* (L.) and despite extensive surveys of other coastal wading birds in Florida and the Gulf of Mexico, neither *I. dimorpha* nor the species described herein has been reported (e.g., Sepúlveda et al., 1994; Spalding et al., 1996; Sepúlveda et al., 1999; Dronen & Chen, 2002; Dronen et al., 2003; Guillén-Hernández et al., 2008; Ortega-Olivares et al., 2011).

**Table 2:** Estimates of evolutionary divergence over mitochondrial cytochrome *c* oxidase I gene sequences between genera (or species if genera were not monophyletic as determined by maximum parsimony or Bayesian inference analysis (Fig. 5) or if a genus was represented by only one species) +/- standard error estimates. Based on an alignment of 586 nucleotides

Group	*Heterospinus mccordi* n. gen. n. sp.
*Andracantha*	0.296 +/− 0.017
*Andracantha sigma*	0.278 +/− 0.018
*Arhythmorhynchus frassoni*	0.318 +/− 0.018
*Bolbosoma*	0.268 +/− 0.017
*Corynosoma*	0.262 +/− 0.017
*Hexaglandula corynosoma*	0.273 +/− 0.018
*Ibirhynchus dimorpha*	0.271 +/− 0.018
*Polymorphus brevis*	0.294 +/− 0.019
*Polymorphus obtusus + P. minutus*	0.312 +/− 0.019
*Polymorphus trochus*	0.273 +/− 0.017
*Profilicollis*	0.321 +/− 0.017
*Pseudocorynosoma*	0.279 +/− 0.017
*Southwellina hispida*	0.279 +/− 0.018
*Tenuisoma tarapungi*	0.282 +/− 0.019

*Examinations of unidentified polymorphid voucher specimens*

None of the voucher specimens labeled *Arhythmorhynchus* (*Southwellina*) sp. examined resembled our specimens. Three individuals from the little blue heron, *E. caerulea,* differed greatly from our specimens, two of them in having a very long trunk and one field of trunk spines; the third was very small (~1 mm) and despite a partially invaginated trunk, showed a field of large spines. The individual from the whooping crane, *G. americana* was a male juvenile whose proboscis and large part of the foretrunk were invaginated but that nevertheless showed large spines on the trunk.

## Discussion

Although historically considered not the best practice, descriptions or re-descriptions of polymorphid genera and species assignment based on the cystacanth stage are not uncommon (e.g., *S. macracanthus*; *Neoandracantha peruensis* Amin & Heckmann, 2017; *Profilicollis chasmagnathi* (Holcman-Spector, Mañé-Garzón & Dei-Cas, 1977); *Corynosoma evae* Zdzitowiecki, 1984; *Pr. rancoensis* Amin, Rodríguez, Farrer, Fierro, Garcés, Rivera & d’Elía, 2023) (Ward & Winter, 1952; Amin & Heckman, 2017; Rodríguez et al. 2017; Hernández-Orts et al., 2022; Amin et al., 2023, respectively). These descriptions are, however, of great value, as cystacanths can be collected more easily than adults, which may be inaccessible due to increasing protections on definitive hosts (Hernández-Orts et al., 2022). This is particularly relevant to polymorphids whose adults mostly infect shorebirds, pinnipeds, and cetaceans (Schmidt, 1985). Such descriptions are possible because cystacanths of polymorphids have been reported as being very similar to adults (Nickol et al., 2002). Indeed, numerous diagnostic morphological characters are visible in cystacanths, including the armature of the proboscis, the size of the neck, the shape of the trunk, the presence/absence/number of spinose fields on the trunk, and the number of cement glands. Although some of these features overlap among polymorphid genera, making morphological identification of these worms particularly challenging, no single genus shares an entire set of such features. Therefore, taxonomic keys for this family, the latest of which is that of Presswell et al. (2020), are still valid and useful. This is the case for the newly collected cystacanths from *P. clarkii* in SC, which, for instance, present a spindle-shaped body (a feature shared with *Neoandracantha* Amin and Heckmann, 2017, *Southwellina*, and *Pseudocorynosoma*), six cement glands (a feature shared with *Andracantha* Schmidt, 1975, *Corynosoma* Lühe, 1904, *Hexaglandula*, *Filicollis* Lühe, 1911, and some species of *Pseudocorynosoma* and *Profilicollis*), and two fields of spines in both sexes (a feature shared with *Diplospinifer* Fukui, 1929, and *Southwellina*). In contrast, these newly collected specimens have very small spines in the posterior spinose field, which sets them apart from individuals of all other genera thus far known in this family. Furthermore, it is now well established that the sole use of diagnostic morphological characters to identify species in the family Polymorphidae can indeed cause taxonomic confusion, as the characters can be so slight and overlooked, and consequently lose their diagnostic value over time as more species are described (Aznar et al., 2006; Presswell et al., 2020). Therefore, it is now critically-important to add molecular and ecological information to descriptions of polymorphids whenever possible. The genetic data obtained in this study supports the erection of the new genus herein. The phylogenetic results of the concatenated LSU and COI gene sequences of *Heterospinus* **n. gen.** suggest that this genus is most closely related to the genera *Ibirhynchus* and *Hexaglandula.* The topology of our trees was similar to those from previous studies (García-Varela et al., 2013; Presswell et al., 2018; Ru et al., 2022) except that the relationships among genera were not resolved, likely due to our shorter LSU sequences. *Heterospinus mccordi* **n. gen.** **n. sp.** sequences appeared sister to the clade containing *I. dimorpha* and *H. corynosoma* sequences, indicating that the newly described species is most closely related to these genera. The genetic distance between our COI sequences and those of other polymorphid genera, however, supports *Heterospinus* **n. gen.** as a distinct genus within the family Polymorphidae.

The intermediate host of the newly collected polymorphid is a crayfish species known to be infected in its native range by the closely-related polymorphid *I. dimorpha*, which we also find in SC (data not shown). *Procambarus clarkii* was introduced in SC about 50 years ago (Pomeroy & Kahl, 1987) and it is not known whether the newly described acanthocephalan species was introduced with its host or if it was acquired locally, both being reasonable assumptions given that polymorphids are successful colonizers (Caballero-Viñas et al., 2021). Further studies are thus needed in the native and non-native ranges of *P. clarkii* to determine whether infection of this crayfish in SC by the newly described polymorphid is a case of host-parasite concurrent introduction or a case of spillover from native or nomadic definitive hosts (Lagrue, 2017). Although the complete life cycle of *Heterospinus mccordi* **n. gen.** **n. sp.** is not known, we may assume that, as for the two other species in this clade whose intermediate hosts are also decapod crustaceans, its definitive hosts are wading birds (García-Varela et al., 2013). This is further supported by evidence that many of the putative definitive hosts are known to forage on crayfish in wetlands, as is the case with white ibis, *E. albus* populations showing positive responses to crayfish abundances in wetlands (Bildstein et al., 1990; Boyle et al., 2014; Cocoves et al., 2021) and known to be a definitive host of *I. dimorpha* (see Schmidt, 1973; García-Varela et al., 2011). Although examination of unidentified polymorphid voucher specimens collected from the little blue heron, *E. caerulea* and the whooping crane, *G. americana* from Florida yielded no identification of the newly described species, these birds, along with the white ibis and the roseate spoonbill, are nevertheless good candidates, and should be examined for infection in coastal SC, as they are common to SC year round (eBird, 2024, https://ebird.org) or as part of their nomadic movements around other parts of their range (Frederick et al., 1996).

In conclusion, a new genus and new species in the family Polymorphidae were erected to accommodate cystacanths collected from the red swamp crayfish, *P. clarkii* outside of its native range. Cystacanths display several morphological characters that overlap with other polymorphid genera when taken individually, but do not match any named polymorphid genus when taken in combination, which confirms the loss of boundaries of diagnostic morphological characters used for this family that other studies have pointed out. The new genus is set apart from other genera in the family in particular by the significantly smaller size of the spines on the second (posteriormost) spinose field on the foretrunk when compared to the size of the spines on the first (anteriormost) spinose field. A small scythe-shaped hook mid-level of the proboscis armature, a single-walled distal end of proboscis receptacle, and a spinose and apparently digitiform bursa are other notable features for these specimens. Furthermore, molecular data generated in this study support a distinct genus and add information to the phylogeny of the family Polymorphidae; the new genus falls into a clade with species of *Hexaglandula* and *Ibirhynchus*, which confirms the previous postulation that species in this clade have decapods as intermediate hosts, and which allows us to assume that the definitive hosts, yet to be discovered, are wading birds.

## Key to the genera of the Polymorphidae (emended key of Presswell et al., 2020)

1. With elongated neck………………………2
2. Without elongated neck……………………3
3. Female proboscis swollen; hooks restricted to short radially arranged rows on anterior surface; 6 kidney-shaped cement glands……………………*Filicollis*
4. Female proboscis not swollen; hooks in long longitudinal rows; 2–6 tubular cement glands……………………*Profilicollis*
5. Trunk claviform or pipe-shaped, proboscis angled towards ventral side……………………4
6. Trunk more or less cylindrical, spindle-shaped or elongated……………………5
7. Two fields of spines on the foretrunk, more or less separated by a bare zone. In piscivorous birds……………………*Andracantha*
8. One field of spines on the foretrunk. In pinnipeds……………………*Corynosoma*
9. Foretrunk with one or two bulbous swellings…………………….6
10. Foretrunk without bulbous swellings……………8
11. Foretrunk with three fields of spines……………………*Neoandracantha*
12. Foretrunk with one or two fields of spines…………….7
13. Foretrunk with one field of spines. In marine mammals……………………*Bolbosoma*
14. Foretrunk with two fields of spines……………………*Diplospinifer*
15. Trunk very elongated, or filiform……………9
16. Trunk not elongated……………………11
17. Hypodermal nuclei spread throughout trunk……………………*Tenuisoma*
18. Hypodermal nuclei restricted to anterior trunk……………………10
19. Anterior field of spines encircles trunk; testes anterior……………………*Arythmorhynchus*
20. Anterior field of spines does not encircle trunk; testes posterior……………………*Ardeirhynchus*
21. Foretrunk of male with two fields of spines……………………12
22. Foretrunk of male with one field of spines……………………13
23. Foretrunk of female with one field of spines; proboscis barrel-shaped……………………*Ibirhynchus*
24. Foretrunk of female with two fields of spines; proboscis cylindrical; Hypodermal nuclei restricted to foretrunk……………………14
25. Genital spines present in males, may be lacking in females……………………*Pseudocorynosoma*
26. Genital spines absent in males and females; Hypodermal nuclei distributed throughout entire trunk…………………….15
27. Foretrunk spines of same size in both fields……………………*Southwellina*
28. Foretrunk spines of different size in both fields……………………*Heterospinus* **n. gen.**
29. With four cement glands……………………*Polymorphus*
30. With six cement glands……………………*Hexaglandula*

## Data Availability

No datasets were generated or analysed during the current study.
